# Pixel to practice: multi-scale image data for calibrating remote-sensing-based winter wheat monitoring methods

**DOI:** 10.1038/s41597-024-03842-8

**Published:** 2024-09-27

**Authors:** Jonas Anderegg, Flavian Tschurr, Norbert Kirchgessner, Simon Treier, Lukas Valentin Graf, Manuel Schmucki, Nicolin Caflisch, Camille Minguely, Bernhard Streit, Achim Walter

**Affiliations:** 1 Plant Pathology Group, Department of Environmental System Science, ETH Zurich, Zurich, 8092 Switzerland; 2 Crop Science Group, Department of Environmental System Science, ETH Zurich, Zurich, 8092 Switzerland; 3 Cultivation Techniques and Varieties in Arable Farming, Plant-Production Systems, Agroscope, Nyon, 1260 Switzerland; 4 Earth Observation of Agroecosystems Team, Division Agroecology and Environment, Agroscope, Zurich, 8048 Switzerland; 5 School of Agricultural, Forest and Food Sciences HAFL, Bern University of Applied Sciences, Zollikofen, 3052 Switzerland

**Keywords:** Environmental impact, Environmental monitoring

## Abstract

Site-specific crop management in heterogeneous fields has emerged as a promising avenue towards increasing agricultural productivity whilst safeguarding the environment. However, successful implementation is hampered by insufficient availability of accurate spatial information on crop growth, vigor, and health status at large scales. Challenges persist particularly in interpreting remote sensing signals within commercial crop production due to the variability in canopy appearance resulting from diverse factors. Recently, high-resolution imagery captured from unmanned aerial vehicles has shown significant potential for calibrating and validating methods for remote sensing signal interpretation. We present a comprehensive multi-scale image dataset encompassing 35,000 high-resolution aerial RGB images, ground-based imagery, and Sentinel-2 satellite data from nine on-farm wheat fields in Switzerland. We provide geo-referenced orthomosaics, digital elevation models, and shapefiles, enabling detailed analysis of field characteristics across the growing season. In combination with rich meta data such as detailed records of crop husbandry, crop phenology, and yield maps, this data set enables key challenges in remote sensing-based trait estimation and precision agriculture to be addressed.

## Background & Summary

Site specific crop management in heterogeneous fields is widely recognized as a promising avenue towards increasing the efficiency and sustainability of agricultural production^1–3^. Timely availability of accurate, spatially explicit information on crop growth and vigor, phenological development, and health status is key for the implementation of such practises^4^. Over the past 10 to 15 years, unmanned aerial vehicles (UAVs) equipped with various lightweight sensors have become instrumental in collecting such data, with ongoing efforts to enhance data interpretation and link remotely sensed proxies to vegetation characteristics^5–11^. A key advantage of UAV-based vegetation monitoring is its temporal and spatial flexibility, along with the high spatial resolution (i.e., ground sampling distance [GSD]) of data products^4^. However, the monitoring area is limited compared to high-altitude platforms such as satellites. Therefore, the transferability of methods to infer plant or vegetation characteristics from remotely sensed signals across scales is of significant interest (see e.g.,^12–14^). High-resolution imagery offers significant potential for calibrating and validating crop trait estimation methods based on remote sensing data^15–18^.

A major challenge in interpreting remote sensing signals in the context of commercial crop production is the large variability in the appearance of crop canopies within and across sites and over - sometimes very short - periods of time. While some of the within-field variability is relevant for designing tailored management interventions^1^, it can also result from soil-related factors, cultivar properties, or management practices *per se*. For example, a single pass with a harrow can significantly alter the appearance of crop canopies instantly, without affecting physiological status or biophysical characteristics. Therefore, robust methods applicable across a broad range of scenarios typically encountered in practise are essential to accelerate the adoption of precision agriculture principles in commercial production.

In the context of this challenge, here we describe a large multi-scale data set comprising (i) nearly 35,000 high-resolution (~2.7 mm to ~8 mm GSD), geo-referenced, aerial RGB images captured from altitudes of 10 m, 30 m, and 50 m; Along with the raw imagery, we provide geo-referenced orthomosaics and digital elevation models as well as shapefiles describing the position of all relevant subareas (field borders, reference areas, treatments); (ii) co-registered ground-based images with a very high spatial resolution (~0.4 mm GSD); (iii) Sentinel-2 satellite imagery with a GSD of 10 m, for nine commercial wheat fields with contrasting management located across the central plains of Switzerland from before tillering to after harvest; finally (iv) we provide rich meta data characterizing each flight and each field.

**Fig. 1:**
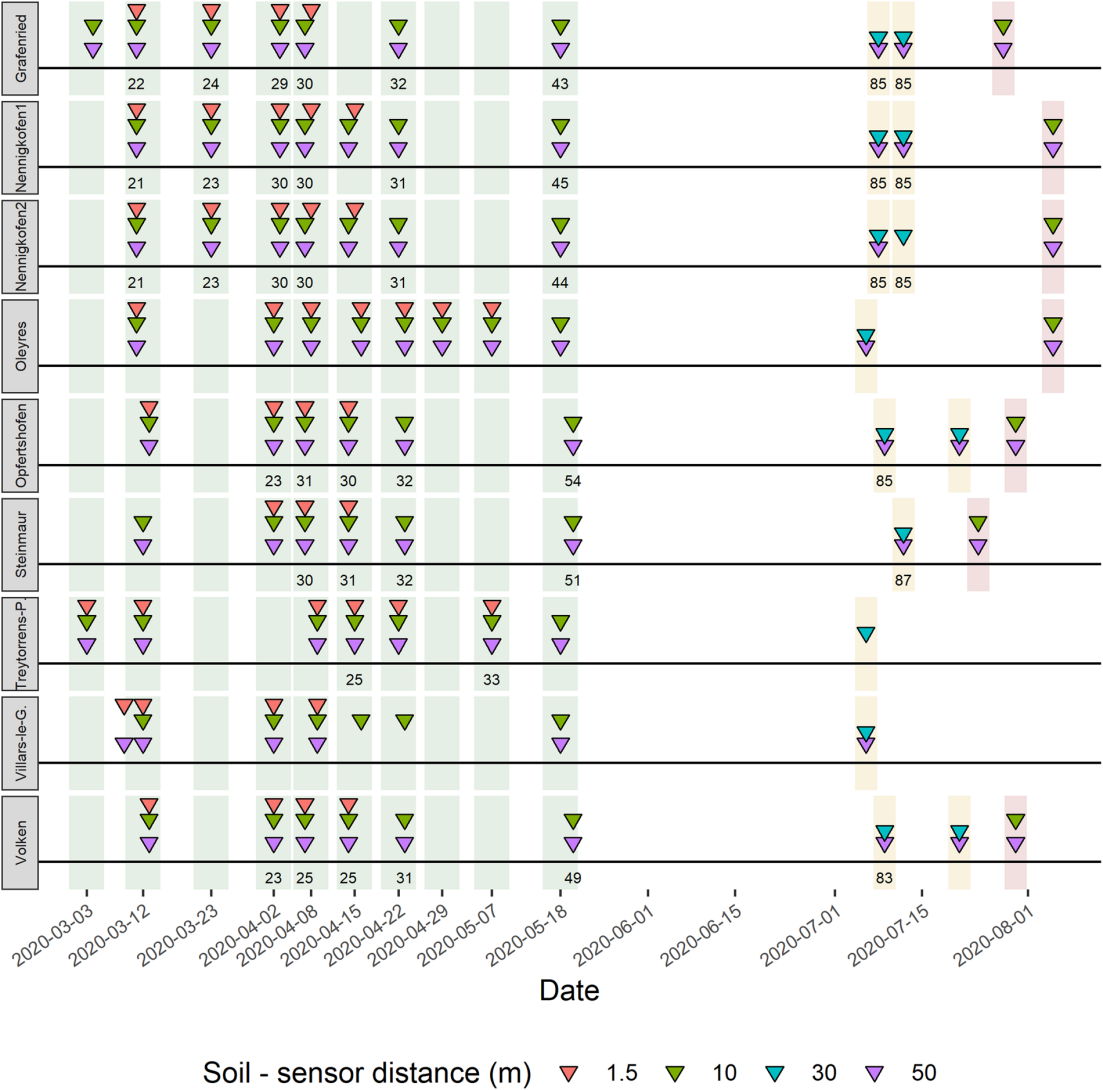
Overview of the measurement campaign that produced the published data set. Nine commercial wheat fields (indicated by the name of the municipality in the grey boxes) were imaged throughout the main growing season of 2020 using hand-held cameras at a distance to the soil of approximately 1.5 m, and unmanned aerial vehicles at different flight altitudes (10 m, 30 m, and 50 m). Measurements during the early growing season can be roughly grouped into measurement events that span 1–3 days (indicated by green vertical bars). Most measurements were performed during early growth stages (GS 20–33), with a last measurement performed shortly before or during heading (GS 44–54; indicated by green vertical bars). An additional 1–2 flights were carried out during maturation (GS 85–87; yellow vertical bars), and a final flight was performed after harvest (red vertical bars) on most sites. Numbers represent the estimated growth stage at the time point of imaging.

The original main purpose of this data set was to assess the feasibility of UAV-based weed monitoring at critical growth stages under a range of environmental and management conditions^7^. Chemical, mechanical, and no weed control measures were applied to subareas of each field. Imaging procedures covered all treatments. An overview of the experimental sites and the measurement campaign is given in Table 1 and Fig. 1. Basic phenology and yield maps are available for some and detailed documentation of crop management is available for all fields. Binary vegetation-soil segmentation masks are provided for ground-based and UAV-based aerial images as a benchmark for the estimation of vegetation cover, along with estimates of their accuracy.

**Table 1:** Overview of the experimental sites included in this study.

Site name	Coordinates (CH1903+/LV95)	Altitude (m.a.s.l.) mean (min ; max)	Wheat cultivar	Sowing date	Field area covered (m^2^) (10 m ; 50 m flight)
Nennigkofen1	2’604’880, 1’225’100	494.2 (492.6; 494.9)	Montalbano	27.10.2019	1712; 10315
Nennigkofen2	2’604’880, 1’225’110	535.5 (534.5; 537.0)	Montalbano	27.10.2019	2061; 10680
Opfertshofen	2’690’780, 1’292’560	609.4 (607.5; 613.7)	Runal	14.10.2019	2705; 27258
Villars-le-Grand	2’567’080, 1’194’570	433.1 (432.7; 433.4)	Hanswin	14.11.2019	1883; 7177
Steinmaur	2’675’830, 1’261’260	458.8 (456.5; 461.2)	Hanswin	16.10.2019	1880; 11008
Volken	2’689’580, 1’270’380	406.4 (403.1; 407.5)	Montalbano	11.11.2019	2900; 8793
Oleyres	2’569’270, 1’190’410	514.5 (511.7; 515.7)	Montalbano	11.11.2019	2271; 16088
Treytorrens-Payerne	2’551’521, 1’180’892	682.9 (680.5; 683.7)	Nara	24.10.2019	2954; 14153
Grafenried	2’605’650, 1’213’620	542.4 (541.6; 542.8)	Hanswin	02.11.2019	1285; 7395

Site altitude is reported as the average, minimum, and maximum altitude above sea level of the ground control point positions. This table was adapted from^7^.

This systematically structured data set (multiple sites, frequent measurements, measurements across scales) and rich meta data allow to address several current challenges related to remote-sensing-based trait estimation and precision agriculture. For example, relationships between remotely-sensed vegetation properties throughout the growing season estimated at different scales, and yield patterns could be explored, which may help improving remote-sensing-based yield predictions (e.g.,^19–21^). Methods for the estimation, interpretation, and improved temporal interpolation of various traits estimated from satellite data could be calibrated and validated using accurate estimates from high-resolution imagery (e.g.,^22^). This is a promising approach especially as such trait retrieval accuracy strongly depends on the phenological stage of the targeted canopy^23^. Similarly, the comparably low accuracy of our benchmark method for vegetation cover estimation in images captured at 50 m altitude highlights the need for approaches that perform better at lower spatial resolution (see e.g.,^24–26^). Furthermore, temporal and spatial stability as well as potential drivers of field heterogeneity could be examined across scales^27^. This may include analyses of the effect of management operations and meteorological conditions on field heterogeneity, as well as spatial correlations with yield. The data set offers the opportunity to calibrate and validate methods across a representative sample of contrasting application scenarios^7^.

## Methods

The nine wheat fields, belonging to eight different farms, are distributed across the central plains of Switzerland. Large differences in soil type and composition as well as weather conditions during the growing season were observed between the experimental sites (Table 1). All experimental fields were imaged using DJI Phantom 4 pro (SZ DJI Technology Co. ltd., Shenzhen, China) with their integrated CMOS RGB sensor (5472  × 3648 pixels), and the areas of the plots used for reference data collection (1 m^2^ reference plots) were additionally photographed at ground level at a distance of about 1.5 m from the ground using a Canon EOS 600D and a Canon EOS 700D (5184  × 3456 pixels). Experimental fields were revisited 6–8 times during the early vegetation period (growth stages [GS] 20–33^28^). Flights were performed, whenever possible, under stable light conditions around noon, though some deviations were unavoidable given the extent of the measurement campaign. At 10 m altitude, the UAV was set to fly at 1 m/s with a front overlap ratio of 90 % and a side overlap ratio of 80 % (~2.7 mm/pixel GSD), covering over 100 m^2^ per flight. Flights at 30 m altitude covered more than 1 ha using the same overlap ratio but at a higher flight speed (2.2 m/s), resulting in a GSD of ~8 mm/pixel. The camera was set to a fixed aperture (F/2.8) for all flights, the ISO was varied according to the conditions but kept to a maximum of 400 and the shutter speed was set to a maximum of 1/1250s.

For further processing and comparison over time, each field was equipped with approximately 30 ground control points (GCP), with a higher density in the area of the 10 m flights and a lower density in the higher altitude flights (30 m and 50 m). The GPS position of the GCPs was measured using a GNSS Differential Global Positioning System (R10, Trimble ltd., Sunnyvale, U.S.A.) with a swipos-GIS/GEO real time kinematic (RTK) correction signal (Federal Office of Topography Swisstopo, Wabern, Switzerland). The acquired images were further processed into digital elevation models (DEM) and geo-referenced orthomosaics using the structure-from-motion-based software Agisoft Metashape Professional 1.5.2 (Agisoft LLC, St. Petersburg, Russia). A detailed list of the processing steps was published previously^7^.

Aerial images were segmented pixel-wise into a vegetation and a background soil fraction based on color properties of pixels, using a random forest classifier^7^. The classifier was trained by sampling training pixels from one randomly selected image per flight, resulting in 3626 instances belonging to 65 images. The sampled training data is included in the data set.

Sentinel-2 satellite images corrected for atmospheric effects (processing level 2A) were obtained from Microsoft Planetary Computer using the open-source Earth Observation data analysis library (EOdal)^29^ for all nine field plots. Only the spectral bands with a GSD of 10 m were downloaded, namely the blue (B02), green (B03), red (B04) and near-infrared (B08) bands. Only Sentinel-2 scenes with a scene-wide cloud cover of less than 10% were acquired to ensure minimal cloud and cloud shadow contamination. In addition, the scene-classification layer (SCL) provided as part of each scene was used to filter out all pixels not classified as “vegetated” (SCL class 4) or “bare ground” (SCL class 5). UAV and Sentinel-2 imagery are provided in Swiss national coordinate reference system (CH1903+ / LV95) and UTM32N, respectively.

## Data Records

**Fig. 2:**
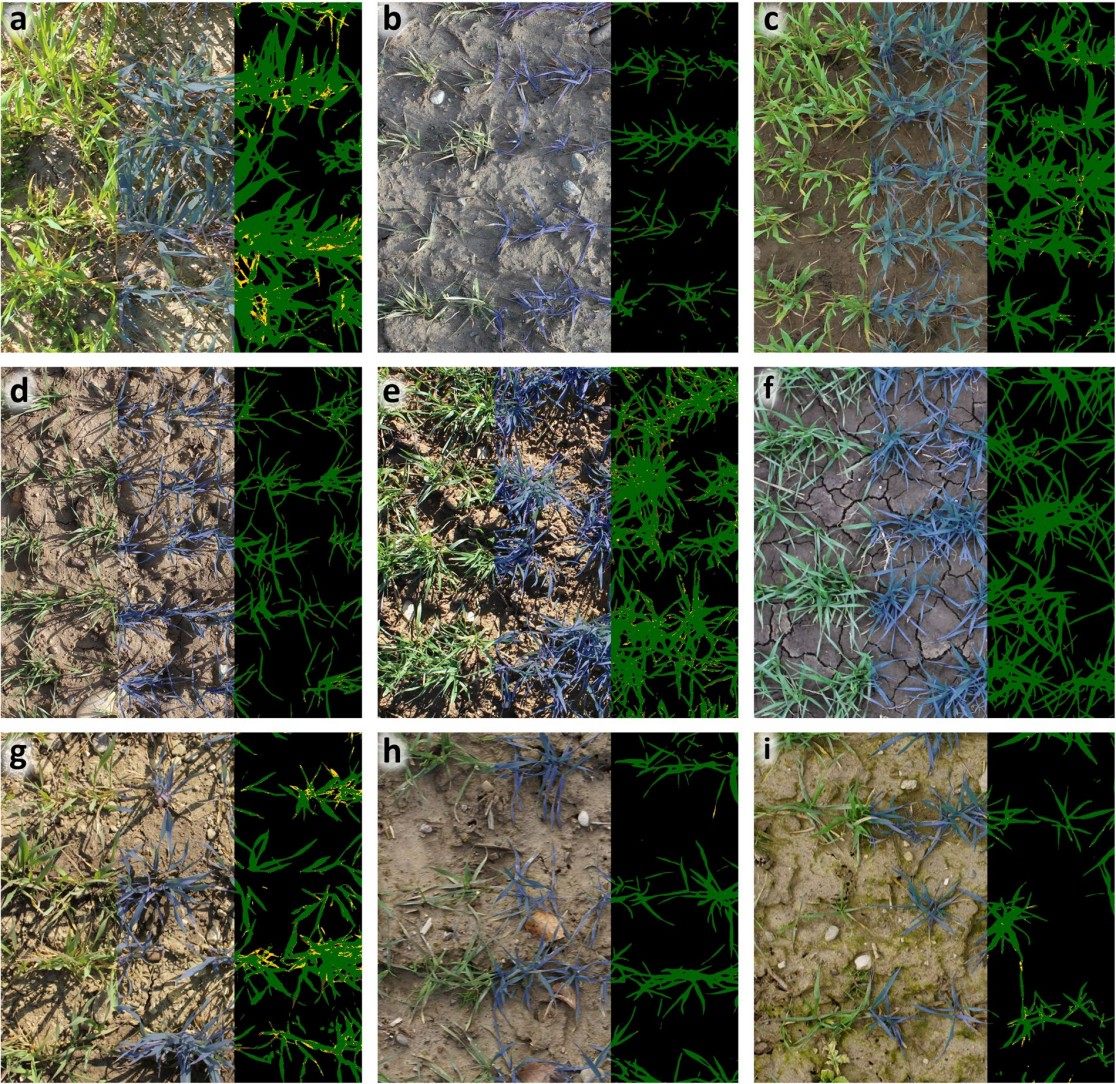
Variability in the appearance of wheat canopies across sites as documented in ground-based high-resolution images at early growth stages. Images are from different measurement dates that correspond to growth stages 20–33 (cf. Fig. 1). The left tile of each image shows the original image, the middle tile is the vegetation - soil mask overlaid on the original image, the right tile shows the final pixel classification into soil background, and green, chlorotic, and necrotic vegetation. (**a**) Nennigkofen1, (**b**) Treytorrens-Payerne, (**c**) Oleyres, (**d**) Volken, (**e**) Opfertshofen, (**f**) Villars-le-Grand, (**g**) Steinmaur, (**h**) Nennigkofen2, (**i**) Grafenried.

The dataset is publicly available from the ETH Zurich publications and research data repository^30^. It is structured first according to data type (‘raw’ - single RGB images, ‘processed’ - orthomosaics and digital elevation models resulting from photogrammetric processing, ‘sat_data’ - Sentinel-2 satellite imagery, and ‘Meta’ - meta information). Within each folder there is one sub-directory per field (see Table 1). These are further structured according to the type of image acquisition (‘10m’, ‘30m’, ‘50m’ or ‘handheld’) and then according to the date of acquisition (YYYYMMDD). The raw data folders also contain the vegetation masks (‘vegmask’; Fig. 2) and the detected sowing rows (for handheld and 10 m flights) for each date.

On the same level, the folder ‘validation’ contains all data that was used for the technical validation of the data set, and all outputs generated during validation. The sub-directories ‘frame_coord_*’ contain the image coordinates of the reference areas; sub-directories ‘handheld’, ‘10m’ and ‘50m’ contain the sampled images and masks for the technical validation and all generated outputs; ‘models’ contains the trained segmentation models.

Several types of meta information can be found in the Meta folder. For each UAV flight, the estimated real-world coordinates of the image corners (‘corners’) are provided, as well as masks for the validation (‘frames’) and the applied weed management treatments (‘treatments’) as .geojson files. Furthermore, for each field, management data can be found. These include sowing dates, variety information, and fertilization or crop protection measures. In addition, yield maps are provided for several fields. Note that these are raw data and may have to be pre-processed according to the needs associated with specific research questions. For each field a .geojson file is provided indicating the covered area for the 10 m and 50 m flight altitude (‘field_mask’). Details on camera parameters can also be found in the meta folder (‘UAV_meta’). A .csv file provides the UAV number used for each flight (three equal models were used) along with an estimate of cloud cover, wind speed, and crop development ratings at the time of the flight. The UAV number refers to available .xml file storing the estimated camera parameters. Finally, ‘UAV_meta’ contains all GCP coordinates. Though all steps involved in photogrammetric processing of the raw imagery have been thoroughly optimised, the available information on the camera parameters and the GCP positions on each field enable this process to be repeated and modified as needed by any qualified person.

## Technical Validation

**Fig. 3:**
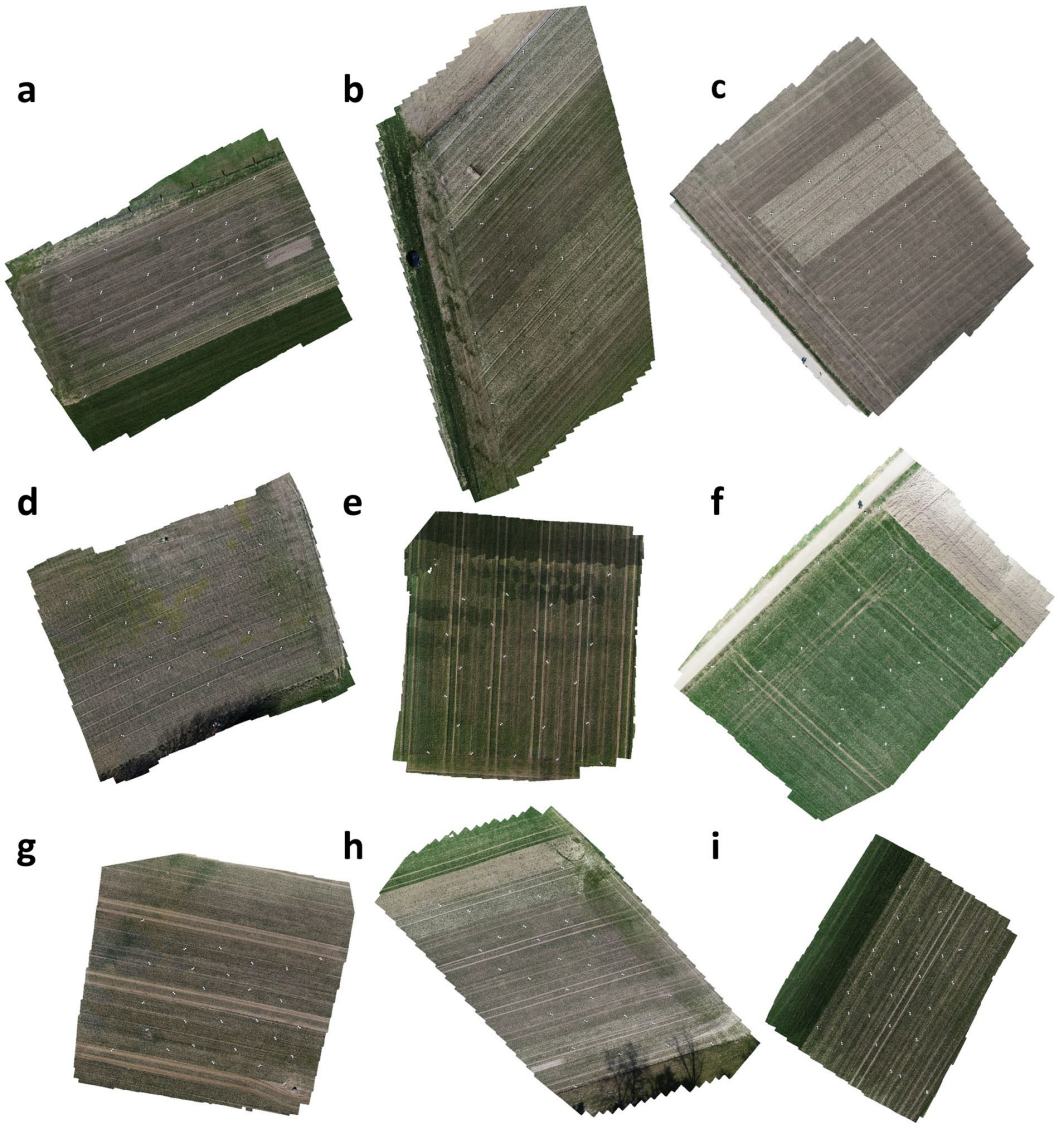
Orthomosaics for all nine experimental sites at early growth stages, based on images captured from an altitude of 10 m. The regularly spaced white dots are the ground control points (GCPs). Note that UAV flights performed at altitudes of 30 m and 50 m cover significantly larger areas, but cover the zones included in the 10 m flights as well. (**a**) Nennigkofen1, (**b**) Treytorrens-Payerne, (**c**) Oleyres, (**d**) Volken, (**e**) Opfertshofen, (**f**) Villars-le-Grand, (**g**) Steinmaur, (**h**) Nennigkofen2, (**i**) Grafenried. This Figure was originally published in^7^.

Visual inspection of the products of photogrammetric processing (orthomosaics) indicated a high spatial accuracy with minor deviations in the GCP and reference area positions across flights and across flight heights for all experimental fields, with absolute x and y errors well below 10 cm. In a few cases, localized inhomogeneities in the orthomosaics related either to changes in light conditions during the flight (see e.g., Fig. 3e) or to abrupt changes of the viewing angles of contributing single images can be observed. Neither of these issues would appear to be problematic as long as single geo-referenced images are used for analyses, which is facilitated here by the availability of real-world corner coordinates for each individual image based on back propagation^31^.

**Fig. 4:**
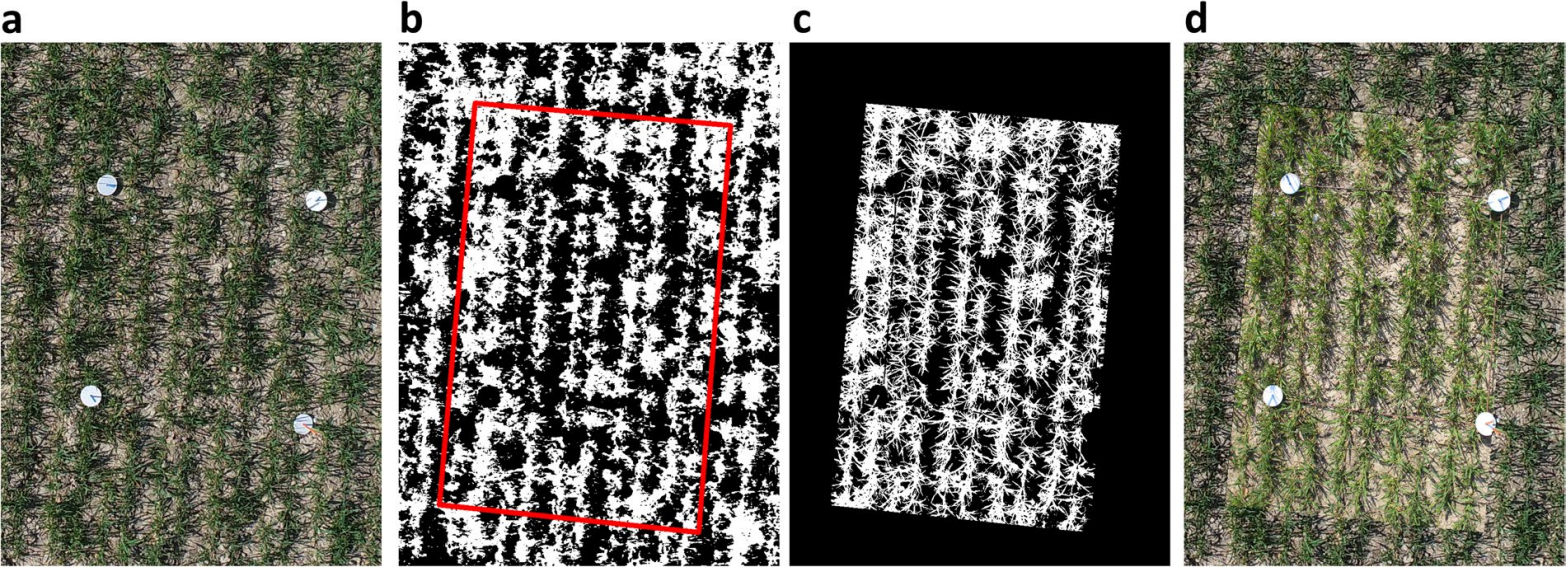
Validation of the vegetation cover estimates based on UAV-based aerial images using very high resolution, ground-based images. (**a**) UAV-based aerial image of a reference area captured from an altitude of 10 m; (**b**) Corresponding binary vegetation-soil segmentation mask, with the area covered by the ground-based high-resolution image indicated by the red frame; (**c**) The registered segmentation mask of the ground-based, high-resolution image; (**d**) Composite image with the registered high-resolution image pasted into the corresponding aerial image. Metrics for the agreement between the two binary masks were done for the entire area covered by the high-resolution image, i.e., the area represented by the red rectangle in (**b**).

**Fig. 5:**
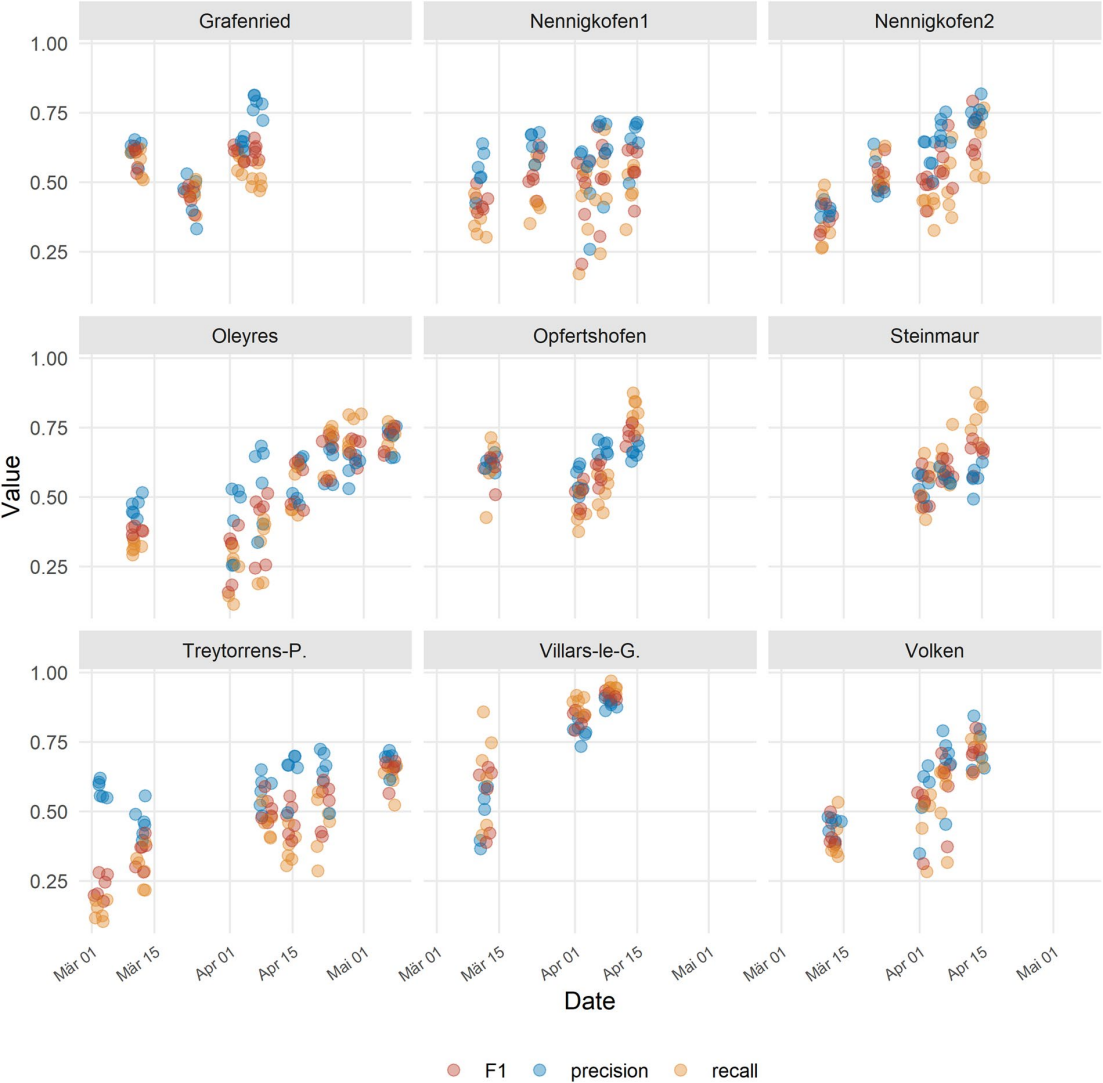
Pixel-level agreement between the vegetation - soil segmentation obtained from the ground-based high-resolution images and UAV-based aerial images captured from an altitude of 10 m. Metrics are reported separately for each of the areas covered by the high-resolution images (approximately 2 m^2^).

**Fig. 6:**
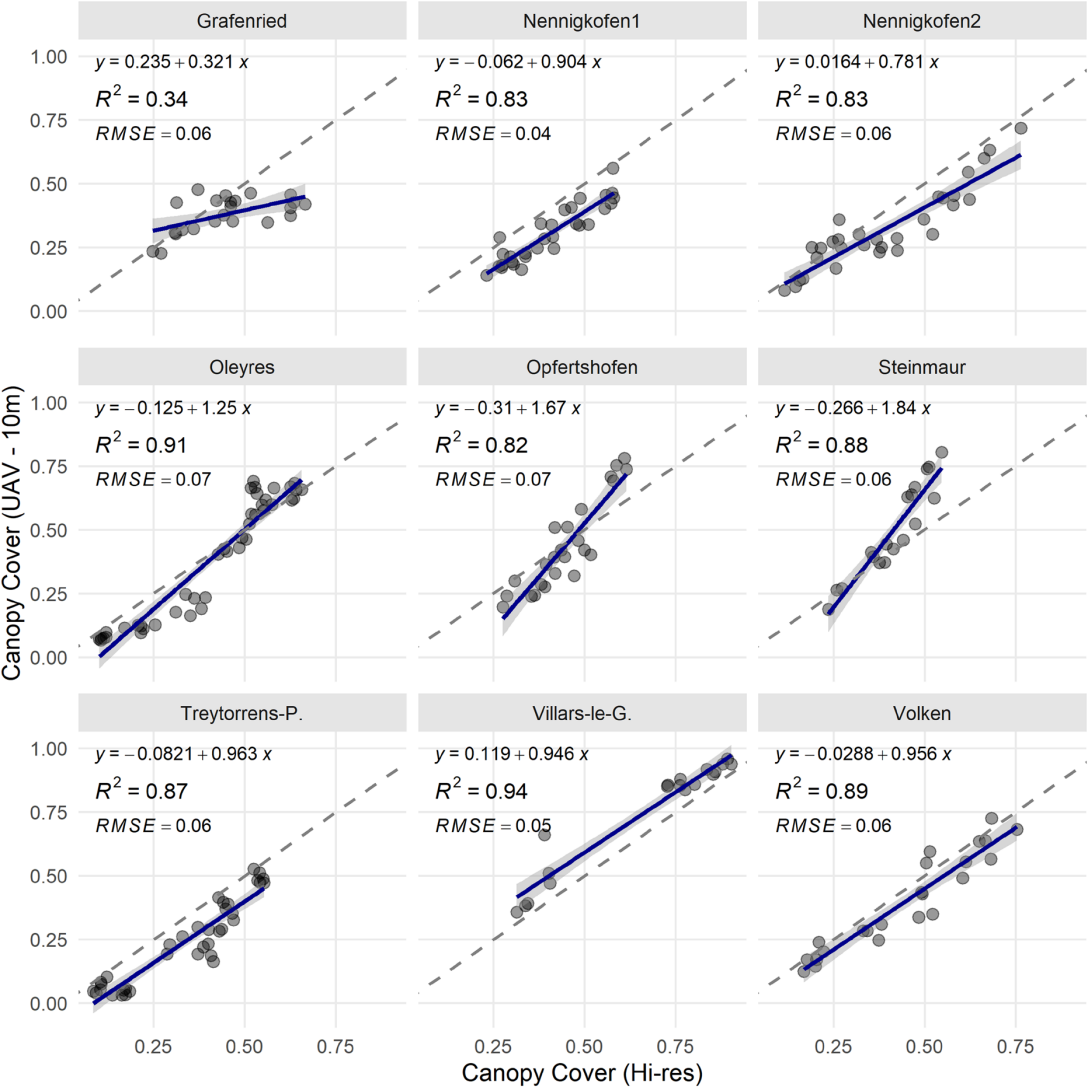
Overall agreement between estimates of vegetation cover obtained from ground-based high-resolution images and UAV-based aerial images captured from an altitude of 10 m. Data from all flights spanning the growth stages 20–54 were pooled at the field level. The dashed line represents the 1:1 line, the blue line represents the least squares line.

To quantify the suitability of the aerial images for estimation of key parameters such as vegetation cover, ground-based high-resolution images were segmented using a deep convolutional neural network (restnet34 - unet++^32,33^) that was trained using the VegAnn dataset^18^ following a previously described procedure^34^. The resulting vegetation fraction in the ground-based high-resolution images was further segmented into a green, chlorotic, and necrotic fraction using a previously trained random forest classifier^34^. This provided an accurate reference value of vegetation cover for the designated reference areas in each field at each time point. Given the high quality of the segmentation (see examples in Fig. 2), they could be used to quantify the accuracy of the UAV-based vegetation cover estimates. For this, we co-registered the high-resolution images and the corresponding segmentation masks to the UAV-based aerial images and their segmentation masks. This was accomplished by first identifying the aerial image providing the most nadir-view for each reference area using their estimated real-world corner coordinates, and then estimating the homography matrix between the high-resolution image and the aerial image based on the four corners of each reference area (Figs. 4, 6). The 3 × 3 homography matrix mapping corresponding points from the source plane to the destination plane was computed by setting up and solving a system of linear equations derived from the four pairs of matching points. Pixel-level metrics for the agreement between the segmentation masks were moderate on average, with F1 values ranging from 0.23 to 0.93 with a mean of 0.55, and from 0.03 to 0.93 with a mean of 0.48, for images taken from 10 m and 50 m flight altitude, respectively (Figs. 4, 5). In contrast, the agreement in overall canopy cover estimates was high for images captured from 10 m flight height (0.82 ≤ R^2^ ≤ 0.94; Figs. 5, 6), except for one field (R^2^ = 0.34). They were, however, only moderate for images captured from an altitude of 50 m (0.18 ≤ R^2^ ≤ 0.89; not shown). For some sites, UAV-based vegetation cover estimates slightly over- or underestimate the actual value as derived from the high-resolution images. The apparent limitations of the benchmark method for estimation of vegetation cover thus highlight the need for improved methods.

## Usage Notes

Additional information, such as weather data to investigate plant growth^35^ or stress indicators^36^ can be obtained either from the Swiss Federal Office of Meteorology via idaweb or from agrometeo for numerous stations throughout Switzerland (see Table 2 for additional sources of potentially useful meta data). The data set is very rich in terms of different soil types, environmental conditions, wheat varieties and management practices, as well as lighting conditions at the time of measurement. Depending on the use case, this variability in the data set can be seen as both an advantage and a disadvantage. On the one hand, the data set can be considered as a representative sample of scenarios that would be encountered in practice^7^, enabling detailed analyses of method robustness. On-farm fields typically have a more complex history and management is more diverse than for fields located on research stations (e.g., types of machinery used). On the other hand, this may lead to cultivar-specific differences across fields that are confounded with differences in environmental (pedo-climatic) conditions across fields, and it may be challenging to disentangle these effects. Also, the data set covers winter wheat fields only, and extrapolation or generalization to other crops may be difficult. Depending on the flight height, the physical resolution may be insufficient for some applications. Especially at early growth stages, individual wheat leaves and young weed plants may be similar in size to a pixel. Mixed soil-plant pixels may therefore cause problems for analyses that focus on object shapes (see Fig. 4 in^7^ for an illustration). Yet, resolution is still much higher than in any satellite-derived product, while the area covered is still large enough to allow for comparisons with data from high-altitude platforms such as satellites. The original purpose of the data set was to create a broad basis for the development and validation of image-based methods for weed mapping in wheat fields. Accordingly, flights targeted the periods preceding complete canopy closure as well as late senescence, when we expected tall-growing weeds with a contrasting phenology to be discernible in dense canopies based on their color. Missing measurements around emergence in autumn and around heading and flowering may represent a limitation for certain use cases such as yield prediction.

**Table 2:** Sources of meta information for further analysis.

Data Type	Description	Access
Weather	Weather station network of the federal office of agriculture	https://www.agrometeo.ch
Weather	Weather data from the federal office of meteorology	https://gate.meteoswiss.ch
Soil information	Data from U.S. Department of Agriculture Natural Resources Conservation Service (USDA-NRCS) and National Cooperative Soil Survey (NCSS) databases	https://CRAN.R-project.org/package=soilDB (R-package)
Elevation	Several web services are available that provide access to elevation data	https://CRAN.R-project.org/package=elevatr (R-package)
Other geographical information	Annual farmer declarations at parcel level and other geographical data	https://geodienste.ch
Other geodata	Maps, Soil, Geology etc.	https://map.geo.admin.ch
Satellite Data	Python library designed to help handling satellite data	https://pypi.org/project/eodal/ (Python library)
Phenology Data	Facilitates the prediction of crop phenological stages through the utilisation of environmental covariate measurements.	https://CRAN.R-project.org/package=DyMEP (R-package)

Where possible we provided a tool that can be implemented directly into software, otherwise we provided URLs.

## Data Availability

A detailed list of photogrammetric image processing steps within Agisoft Metashape Professional 1.5.2 (Agisoft LLC, St. Petersburg, Russia) was published previously^7^ and is freely available from the article website. Python code allowing to reproduce the results of the technical validation is open-sourced at https://github.com/and-jonas/wheat-field. Specifically, we share our methods for (i) image segmentation using pixel-wise color-based classification and deep-learning based segmentation for UAV and handheld images, respectively; (ii) retrieval of UAV images for specific sub-areas of interest on a field, such as the reference areas for which high-resolution images are available; (iii) downloading and filtering of the satellite data, and (iv) co-registration of high-resolution handheld images and UAV images to compare image processing outputs across scales. Thus, the code also illustrates how provided meta data can be used to subset images, for example.
